# The “drive to eat” hypothesis: energy expenditure and fat-free mass but not adiposity are associated with milk intake and energy intake in 12 week infants

**DOI:** 10.1093/ajcn/nqab067

**Published:** 2021-04-13

**Authors:** Jonathan C Wells, Peter S Davies, Mark Hopkins, John E Blundell

**Affiliations:** Childhood Nutrition Research Centre, Population, Policy and Practice Research and Teaching Department, University College London Great Ormond Street Institute of Child Health, London, United Kingdom; Child Health Research Centre, Centre for Children's Health Research, University of Queensland, South Brisbane, Australia; School of Food Science and Nutrition, University of Leeds, Leeds, United Kingdom; Appetite Control and Energy Balance Research Group, School of Psychology, Faculty of Medicine and Health, University of Leeds, Leeds, United Kingdom

**Keywords:** appetite control, energy intake, fat-free mass, fat mass, energy expenditure, infant

## Abstract

**Background:**

Recent work has challenged the long-held assumption that appetite functions to maintain stable body mass and fat mass (FM), suggesting instead that appetite matches food intake to energy expenditure and its correlate, fat-free mass (FFM). Whether this scenario applies to young infants, in chronic positive energy balance, remains unknown.

**Objectives:**

To test associations of components of energy expenditure and body composition with milk intake (MI) and energy intake (EI) in 12-week infants, by reanalyzing published cross-sectional data.

**Methods:**

Data were available for 48 infants. In addition to anthropometric measurements, we assessed MI and EI by test-weighing, sleeping metabolic rate (SMR) by indirect calorimetry, and FFM, FM, and total energy expenditure (TEE) by doubly labeled water. Mean parental height was calculated as a marker of infant growth drive. Correlation and multiple regression analyses were applied.

**Results:**

MI and EI correlated with FFM (r = 0.47 and 0.57, respectively; *P* < 0.01), but not FM (*P* > 0.6). MI and EI correlated with SMR (r = 0.42 and 0.53, respectively; *P* < 0.01) and TEE (r = 0.50 and 0.49, respectively; *P* < 0.01). SMR and TEE correlated with FFM (r = 0.41 and 0.42, respectively; *P* < 0.01), but not FM (*P* > 0.2). In a multiple regression analysis, MI was independently associated with TEE (partial r = 0.39) and FFM (partial r = 0.35). EI showed similar associations. Mean parental height was correlated with weight gain, MI, and EI.

**Conclusions:**

As in adults, MI and EI in young infants were strongly associated with FFM and with total and sleeping components of energy expenditure, but not with fatness. The infant's growth drive contributed to these associations. This suggests that appetite is regulated by the rate of energy expenditure, the size of energy-using tissues, and tissue deposition rate, and that the high levels of body fat characteristic of infants may not constrain weight gain.

## Introduction

For many decades, research on energy intake (EI) and energy expenditure (EE) was conducted with little cross-talk (1). In adults, it was widely assumed that the biological function of appetite is to match EI against EE, to maintain constant body mass or fat stores. The idea of “lipostasis” was postulated by Kennedy (2) 70 years ago, on the basis of studies on rats made hyperphagic through lesions in the ventromedial nucleus of the hypothalamus. This approach, attributing excess weight gain and obesity to perturbation of the adipose regulatory system, appeared confirmed by the discovery of leptin (3), but critical evidence linking appetite control to body fat has not been forthcoming. An alternative approach to appetite control was also proposed several decades ago. Based on studies of EI and EE in army cadets, Edholm et al. (4) argued that differences in food intake must originate in differences in EE. Although plausible, the concept was neglected for over 50 years.

The idea that EI arises from EE has several consequences. First, it implicates the resting metabolic rate (RMR), as this accounts for 60–70% of total EE. Second, this implicates fat-free mass (FFM), as this component of body composition accounts for ∼65% of variability in RMR, and fat mass (FM) only accounts for ∼7%. Support for Edholm et al.’s (4) approach was obtained in adults by a study showing that EI correlated with FFM but not FM (5), a finding replicated in participants under various dietary conditions (6, 7), including in adolescents (8). RMR is also closely associated with EI (9, 10) and has been shown to mediate the effect of FFM on EI (11).

These findings have led to a formal statement of the current theory, in which RMR “represents a physiological source of hunger that drives food intake at a level proportional to basal energy requirements. This long-term (tonic) signal of energy demand would help ‘tune’ EI to EE, and help ensure the maintenance and execution of key biological and behavioral processes” (12). Importantly, vital organs collectively account for 60–70% of RMR in humans, though only ∼6% of weight (13, 14). This approach therefore considers that EE at the level of individual tissues and organs imposes a “draw” on appetite, which acts on EI to meet ongoing metabolic needs. Should the EE of organs and tissues change—for example, through greater physical activity or somatic growth—appetite is predicted to respond by stimulating increased EI. However, physical activity appears to correlate more weakly with EI than does RMR (15).

In contrast to FFM, FM appears to have a limited mass-action effect on appetite. FM may generate a weak positive effect on EI, since it makes a small contribution to RMR, but it also has a stronger inhibitory effect through the well-described leptin signaling pathway (16–18). However, as FM accumulates, the body tends to become resistant to leptin, and the “braking” effect of FM loses its efficacy (11, 19). An intriguing issue is whether these associations are replicated in early life. Young infants are characterized by a chronic positive energy balance, high body fatness, and relatively low levels of physical activity (20). We investigated associations of milk intake (MI) and EI with FFM, FM, and EE components, reanalyzing data from a study of infants aged 12 weeks (21).

## Methods

We recruited 50 healthy, full-term infants into a comprehensive study on energy metabolism from the Rosie Maternity Hospital, Cambridge, over the period 1992–1993. Half the sample had been exclusively fed formula from 2 weeks or earlier, whereas the other 25 had been exclusively breastfed at birth and remained predominantly breastfeeding at 12 weeks. In the original study, the exposure was infant feeding mode and the primary outcomes were body composition and EE. The data have been reported previously (21, 22).

In each group, supplementary foods could have been introduced after 11 weeks, provided that their contribution to total EI was minimal. Infants below the 10th or above the 90th Gairdner Pearson percentiles for birth weight were excluded. Ethical permission for the project was granted by Cambridge Health Authority and the Medical Research Council's Dunn Nutrition Unit. At 12 weeks of age, a number of measurements were made in the infant's home over a period of 1 week (days 1 to 7). These included anthropometry, MI, body composition, and sleeping and total EEs. Maternal education was categorized as completing either secondary school or further (university) education. The height of both parents was obtained by self-report.

### Anthropometry

Measurements of infant weight were made on days 1 and 7, and of supine length on day 1. These data were used to calculate the BMI and weight gain over the study week. Weights were obtained with the infant nude using Seca 724 electronic scales accurate to 20 g. Supine infant length was measured to the last completed millimeter using a Harpenden infantometer (Holtain, Dyfed).

### Offspring growth drive

In the absence of genetic data, we used parental height to provide a proxy marker of infant growth drive. Twin studies have demonstrated high heritability (∼80%) of postnatal weight gain (23). In addition, previous studies of large samples have reported similar correlations of maternal and paternal height with infant weight gain (24, 25). We therefore assumed that mean parental height would act as a robust marker of an infant's growth drive.

### Milk intake and total energy intake

In the breastfed infants, MI was determined by test-weighing over two 24-hour periods using an electronic balance (Sartorius 3862 MP8–1) accurate to 0.1 g and programmed to average 10 consecutive weights over a few seconds. Because there is a systematic error from test-weighing, due to insensible water loss from the lungs and skin during feeds, milk intake was corrected for this using a rate of 0.03 g/kg/min (26), multiplied by the duration of the feeds. In formula-fed infants, MI was measured by weighing formula bottles before and after feeds over two 24-hour periods. This was undertaken using electronic scales accurate to 1 g (Bronso Uni-scale, PC International Ltd).

In both groups, intakes of nonmilk water and supplementary foods were also recorded on the same scales, or by volume where appropriate. The energy content of the foods was taken from manufacturers’ information. These data were used to estimate EI from nonmilk sources, assuming a digestibility of supplementary foods of 50%.

The energy provided by milk was calculated from the data on MI, assuming that the energy density of breast milk was 259 kJ/100 ml (27) and that of formula milk was 276 kJ/100 ml. The energy from supplementary food intake was added to give values for total EI based on these intake measurements.

### Sleeping metabolic rate

The basal metabolic rate (BMR) cannot be measured in infants for obvious ethical reasons concerning fasting; using the RMR is also inappropriate, as it is not possible to prevent physical activity. The sleeping metabolic rate (SMR) was therefore measured in the home of the infant on 1 occasion during the study week. However, it is important to note that in adults the SMR is lower than the BMR and includes the energy cost of arousal (28). Therefore, SMR represents a practical marker of RMR in infants but is not directly comparable to other age groups.

Oxygen consumption and carbon dioxide production were measured using a Deltatrac MK1 metabolic monitor (Datex, Helsinki). The infant was allowed to fall asleep as normal in a sealed 50-liter plastic cot, and respiratory gas exchange was measured for 60 minutes or until the infant awoke (minimum time 20 minutes). The temperature in the cot was monitored for safety purposes, but not controlled. Energy expenditure was calculated using Weir's equation (29), which has been used successfully in validation studies comparing EE in young infants by different measurement techniques (30, 31). SMR was defined as the mean EE over the entire measurement period. To minimize any contribution of the costs of digestion to SMR, the minimum observed EE was calculated as the lowest mean EE over any 5 consecutive minutes within the measurement period (32), and was used in subsequent analyses.

### Total EE and body composition

Total EE and body composition were measured using doubly labeled water. The details of our methodology have been described previously (33, 34). Briefly, urine samples were collected daily for 7 days after the administration by mouth of a dose of ^2^H_2_^18^O. Doses were intended to give 0.28 g of ^18^O and 0.10 g of ^2^H per kg body weight. Urine samples were obtained by leaving cotton wool balls in the diapers of the infants during the daytime, but not during the night. The parents were asked to check the diaper frequently for urination, and the time of voiding was taken as the midpoint between the last 2 times of checking. In the calculation of EE, the fractionation factors were those used by Lifson et al. (35) in their original experimental work, and the proportion of water subject to fractionation was taken to be 0.15 (36). The respiratory quotient was assumed to be 0.85 based on measurements of food quotients in approximately 200 infants (37). Isotope dilution spaces were calculated by the back extrapolation method (38).

The oxygen dilution space was divided by 1.01 to give a value for total body water (39). This value was then adjusted for the water content of lean tissue to give FFM, using age- and sex-specific hydration values from the reference child of Fomon et al. (40). These values were 80.0% (boys) and 79.9% (girls) at 3 months. FM was then calculated as the difference between FFM and weight.

### Hypotheses

In keeping with adult data (7), we hypothesized that FFM, SMR, and TEE would all correlate with MI and EI, and that FM would not show such correlations. In exploratory analyses, we tested whether weight gain over the study week was associated with baseline FM. We also tested whether infant weight gain, MI, and EI were associated with mean parental height, which would indicate an influence of genetic growth drive on appetite.

### Sample size and statistical analyses

The original study was intended to compare metabolic variables between breastfed and formula-fed infants. The sample size of 25 per group was adequate to identify differences equivalent to 0.8 SDs in magnitude. The sample size available for analysis here (*n* = 46) can detect correlations between variables of r > 0.4, with a 2-tailed type 1 error rate α of 0.05 and a type 2 error rate ß of 0.20.

We tested for skewness and kurtosis, and ran Shapiro-Wilks tests, also investigating QQ-plots. Based on these assessments, all variables could be considered normally distributed except for weight gain, which was right skewed and remained so even after natural log transformation. We therefore used Pearson's correlation analysis and a graphic analysis to explore bivariate associations between all variables except weight gain, where we used Spearman's correlations. We used 1-sided tests to investigate correlations between FFM, SMR, TEE, EI, MI, weight gain, and mean parental height, as we predicted positive associations between these variables. We used 2-sided tests to investigate correlations of FM with other variables, as we expected null associations.

 We selected exposures and outcomes that made minimal use of the same raw data, in order to avoid a common error contributing to the correlations. We used the Benjamini-Hochberg method to correct for multiple comparisons (41). Applying this procedure, correlations were considered significant at a *P* value < 0.025. We further constructed multiple regression models to test independent associations of body composition and EE variables with MI and EI. We averaged parental heights to provide a marker of “growth drive” in the infant. All analyses were conducted in SPSS version 24 (Statistical Product and Service Solutions; IBM Corp).

## Results

**FIGURE 1:**
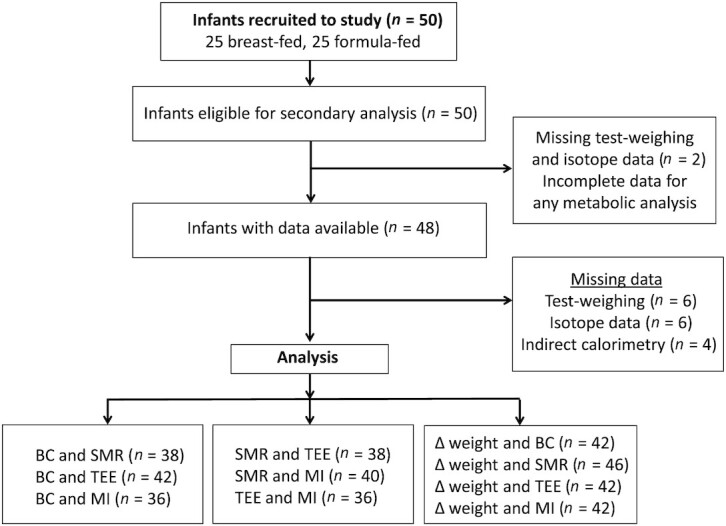
Flow diagram of study. Abbreviations: BC, body composition; MI, milk intake; SMR, sleeping metabolic rate; TEE, total energy expenditure; ∆ weight, weight gain per week.

Among the original sample of 50 infants, missing data points in infants were due to failing to complete a successful isotope dosing (*n* = 8), an indirect calorimetry measurement (*n* = 4), or test weighing over 2 complete 24-hour periods (*n* = 8). After excluding 2 infants who lacked adequate data for any metabolic analysis, the availability of data in the remaining 48 infants is shown in **Figure 1**. Characteristics of those infants with available data at the time of recruitment are given in **Table 1**. Levels of maternal education were high, with all mothers having completed secondary school and 29 also having completed a university degree. All mothers self-identified as of white European ethnicity. Two mothers did not provide their body weight by recall. There were no differences in age, weight, body composition, TEE, SMR, MI, or total EI between breastfed and formula-fed infants (*P* > 0.05 in all cases), or between those included in these analyses versus those missing data (data not shown).

**TABLE 1:** Characteristics of the infants and parents in the sample

	*n* ^1^	Mean ± SD	Range
Parental characteristics			
Maternal BMI, kg/m^2^	46	23.1 ± 3.3	17.8–30.2
Maternal height, cm	48	162.9 ± 6.2	152–178
Paternal height, cm	46	178.5 ± 6.8	161–190
Infant characteristics			
Breast-fed^2^	24	50	—
Male^2^	22	46	—
Birth weight, kg	48	3.54 ± 0.36	2.70–4.17
Gestational age, weeks	48	40.1 ± 1.3	37.0–42.0
Age at study, weeks	48	12.3 ± 0.7	11.0–14.3
Weight, kg	48	5.97 ± 0.64	4.74–7.52
Length, cm	48	61.1 ± 1.7	57.9–64.2
Fat-free mass, kg	42	4.43 ± 0.44	3.39–5.56
Fat mass, kg	42	1.53 ± 0.50	0.61–2.86
Sleeping metabolic rate, kJ/d	44	1313 ± 156	906–1677
Total energy expenditure, kJ/d	42	1922 ± 363	1289–2825
Milk intake by test-weighing, g/d	42	895 ± 164	562–1244
Supplementary energy intake, kJ/d	48	84 ± 119	0–418
Total energy intake, kJ/d	42	2483 ± 462	1486–3383

1 Number of valid data points.

2 As a proportion of the 48 infants.

**FIGURE 2:**
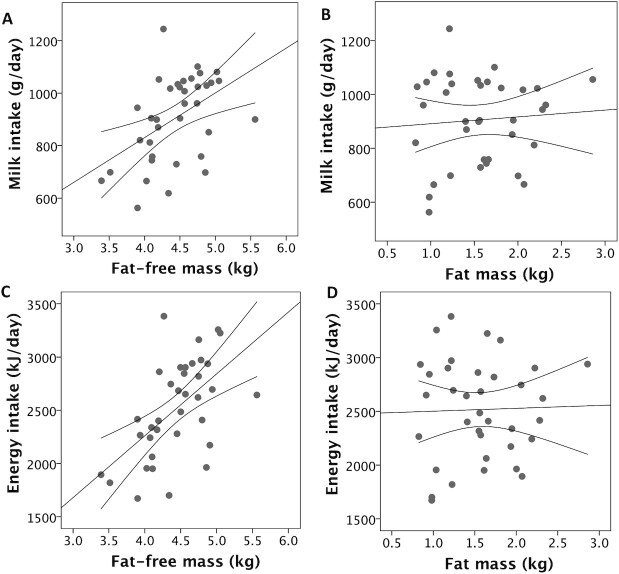
Associations of (A) infant fat-free mass (r = 0.47; *P* = 0.002) and (B) fat mass (r = 0.08; *P* = 0.6) with milk intake, and (C) fat-free mass (r = 0.57; *P* < 0.0001) and (D) fat mass (r = 0.03; *P* = 0.8) with total energy intake. Correlation values are Pearson's coefficients, tests are 1-sided for fat-free mass and 2-sided for fat mass. In all plots, *n* = 36. Error lines provide 95% CIs for the linear associations.

**FIGURE 3:**
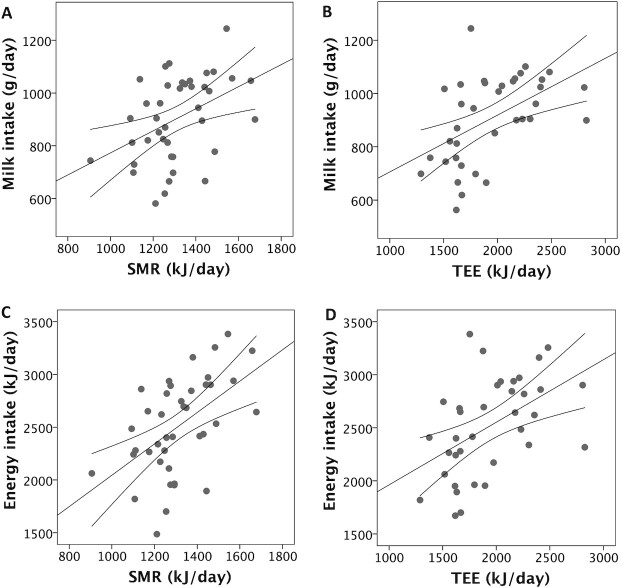
Associations of (A) SMR (r = 0.42; *P* = 0.003) and (B) TEE (r = 0.50; *P* = 0.001) with milk intake, and (C) SMR (r = 0.53; *P* < 0.0001) and (D) TEE (r = 0.49; *P* = 0.001) with total energy intake. Correlation values are 1-sided Pearson's coefficients. *n* = 40 for SMR, *n* = 36 for TEE. Error lines provide 95% CIs for the linear associations. Abbreviations: SMR, sleeping metabolic rate; TEE, total energy expenditure.

**FIGURE 4:**
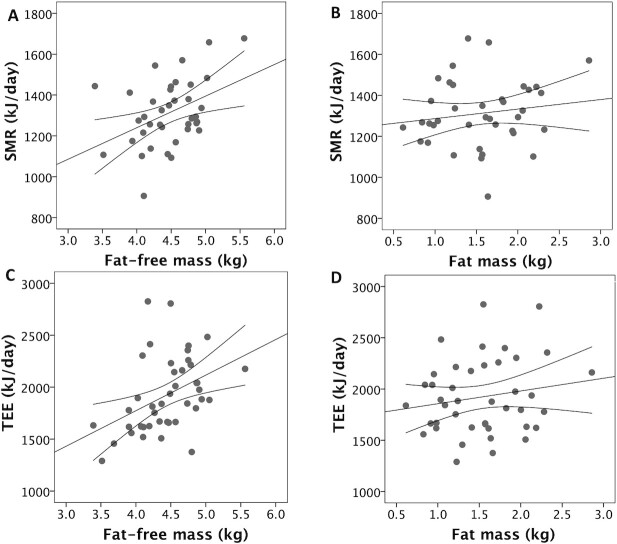
Associations of (A) infant fat-free mass (r = 0.41; *P* = 0.005) and (B) fat mass (r = 0.15; *P* = 0.3) with SMR, and (C) fat-free mass (r = 0.42; *P* = 0.003) and (D) fat mass (r = 0.17; *P* = 0.2) with TEE. Correlation values are Pearson's coefficients; tests are 1-sided for fat-free mass and 2-sided for fat mass. *n* = 38 for SMR, *n* = 42 for TEE. Error lines provide 95% CIs for the linear associations. Abbreviations: SMR, sleeping metabolic rate; TEE, total energy expenditure.

**FIGURE 5:**
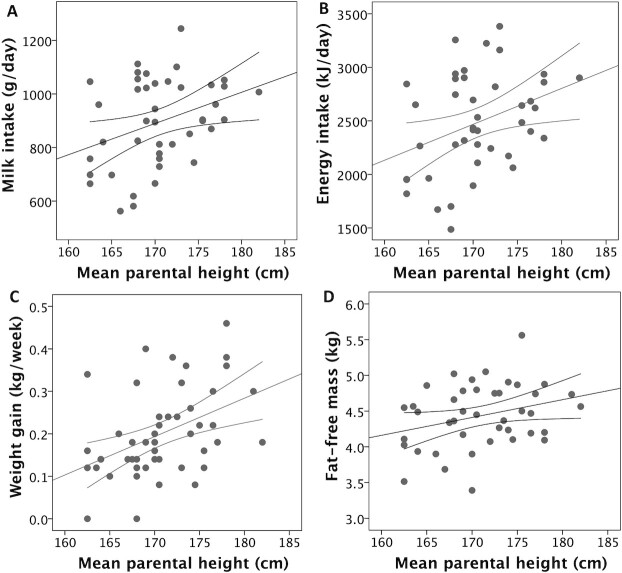
Associations of mean parental height with (A) milk intake (r = 0.35; *P* = 0.012), (B) total energy intake (r = 0.35; *P* = 0.011), (C) weight gain over the study week (r = 0.41; *P* = 0.001), and (D) baseline fat-free mass (r = 0.30; *P* = 0.027). Correlation values are 1-sided Pearson's coefficients, except for weight gain, where a 1-sided Spearman's coefficient is given due to nonnormal distribution of this variable. *n* = 42 for milk intake, energy intake, and fat-free mass; *n* = 48 for weight gain. Error lines provide 95% CIs for the linear associations.

**Table 2** illustrates correlations between body composition variables; markers of EE, MI, and EI; weight gain over the study week; and mean parental height. Scatter plots are also presented in relevant figures. Due to missing data as described in Figure 1, the sample sizes for these correlations varied, ranging between 36 and 48. MI and EI were positively correlated with FFM (*P* < 0.0025 in both cases), but not with FM (**Figure 2**). MI and EI were both positively correlated with SMR and TEE (*P* < 0.005; **Figure 3**). FFM was positively correlated with SMR and TEE (*P* < 0.01), whereas FM was not (**Figure 4**). MI and TEE (*P* < 0.016), but not EI (*P* = 0.05), were positively correlated with weight gain over the study week. FM was not correlated with weight gain (r = −0.26; *P* = 0.1). Mean parental height was correlated with EI, MI, and weight gain (*P* < 0.015), whereas its association with FFM was not significant after correcting for multiple comparisons (r = 0.30; *P* = 0.027; **Figure 5**).

**TABLE 2:** Correlations between body composition, metabolic and parental traits

	FM	SMR	TEE	Milk intake	Energy intake	Weight gain	Parental height
FFM	−0.03	0.41	0.42	0.47	0.57	0.10	0.30
	*P* = 0.8	*P* = 0.005	*P* = 0.003	*P* = 0.002	*P* < 0.0001	*P* = 0.2	*P* = 0.027
	*n* = 42	*n* = 38	*n* = 42	*n* = 36	*n* = 36	*n* = 42	*n* = 42
FM	—	0.15	0.17	0.08	0.03	−0.26	0.05
		*P* = 0.3	*P* = 0.2	*P* = 0.6	*P* = 0.8	*P* = 0.1	*P* = 0.7
		*n* = 38	*n* = 42	*n* = 36	*n* = 36	*n* = 42	*n* = 42
SMR	—	—	0.31	0.42	0.53	0.02	0.01
			*P* = 0.029	*P* = 0.003	*P* < 0.0001	*P* = 0.4	*P* = 0.4
			*n* = 38	*n* = 40	*n* = 40	*n* = 46	*n* = 46
TEE	—	—	—	0.50	0.49	0.36	0.25
				*P* = 0.001	*P* = 0.001	*P* = 0.009	*P* = 0.053
				*n* = 36	*n* = 36	*n* = 42	*n* = 42
Milk intake	—	—	—	—	0.94	0.34	0.35
					*P* < 0.0001	*P* = 0.015	*P* = 0.012
					*n* = 42	*n* = 42	*n* = 42
Energy intake	—	—	—	—	—	0.26	0.35
						*P* = 0.05	*P* = 0.011
						*n* = 42	*n* = 42
Weight gain	—	—	—	—	—	—	0.45
							*P* = 0.001
							*n* = 48

Parental height is the average of maternal and paternal heights. All correlations are Pearson's coefficients, except for weight gain, where Spearman's coefficients are reported. We used 1-sided tests, except for correlations including fat mass, where 2-sided correlations were used. Using the Benjamini-Hochberg procedure to correct for multiple comparisons, correlations were considered significant if *P* < 0.025. Abbreviations: FFM, fat-free mass; FM, fat mass; SMR, sleeping metabolic rate; TEE, total energy expenditure.

Building on the findings described in Table 2, we constructed multiple regression models for the association of MI with TEE or SMR, in each case adjusting for FFM (**Table 3**). MI was associated with SMR independent of FFM; however, the association between FFM and MI was not significant. MI was associated with SMR (partial r = 0.35) independent of FFM, whereas the association between FFM and MI (partial r = 0.29) was not significant. MI was independently associated with both TEE (partial r = 0.39) and FFM (partial r = 0.35).

**TABLE 3:** Multiple regression models of independent associations of milk intake and total energy intake with markers of energy expenditure and fat-free mass

	*n*	Beta	SE	*P*	r^2^	Partial r
Milk intake, g/d						
Constant	34	55.2	251.8	0.8	0.242	—
Sleeping metabolic rate, kJ/d	—	0.32	0.15	0.048	—	0.349
Fat-free mass, kg	—	98.7	58.2	0.100	—	0.291
						
Constant	36	72.3	229.2	0.7	0.304	—
Total energy expenditure, kJ/d	—	0.16	0.06	0.019	—	0.394
Fat-free mass, kg	—	117.9	55.1	0.040	—	0.347
						
Total energy intake, kJ/d						
Constant	34	−442.7	651.9	0.5	0.380	—
Sleeping metabolic rate, kJ/d	—	1.04	0.40	0.015	—	0.421
Fat-free mass, kg	—	364.8	150.7	0.021	—	0.399
						
Constant	36	−247.1	611	0.6	0.378	—
Total energy expenditure, kJ/d	—	0.38	0.17	0.033	—	0.361
Fat-free mass, kg	—	454.7	147.1	0.004	—	0.474

Table 3 also presents results for the regression of total EI on the markers of EE and FFM. Both FFM (partial r = 0.42) and SMR (partial r = 0.40) were independently associated with EI, while in a separate model TEE (partial r = 0.36) and FFM (partial r = 0.47) were independently associated with EI.

## Discussion

This analysis replicates recent work in adults (7), showing that both FFM and EE are associated with milk intake and total EI in 3-month-old infants, whereas FM is uncorrelated with any of these variables. The results shown here support the proposition that FFM and RMR are the strongest determinants of EI, as stated in a recent editorial in this journal (42), and that this enables EI to match the energy demands of the growing infant. In children and adults, studies have provided abundant evidence that excess weight gain, primarily comprising FM, does not inhibit appetite sufficiently to prevent weight gain from continuing (43–45). Our data reproduce this finding by deliberately focusing on an early stage of development, when substantial fat deposition is the norm.

In early life, EI must fund both EE and weight gain. Unlike in older age groups, the energy costs of growth (synthesis and tissue content) account for an unusually high proportion of metabolizable EI in early infancy. At 3 months, for example, on average only ∼15% of total metabolizable EI is allocated to physical activity, ∼45% to basal metabolism, and ∼5% to thermogenesis, while ∼35% (representing both the energy costs of synthesizing new tissue and the energy content of that tissue) is allocated to growth (20). Moreover, infants naturally have high levels of body fat. In this sample, for example, 25.4% (SD, 6.7%) of weight comprised fat, while our new reference data, based on isotopic measurements of body water, show that in both sexes, fat accretion accounts for ∼33% of the weight gained between 3 and 4 months of age (46). Nevertheless, that indicates that two-thirds of weight gain in this age group is comprised of FFM. In a longitudinal study where body composition was measured by deuterium dilution at 3 and 6 months in 38 infants, the proportion of weight gain that was FFM averaged 61% (95% CI: 56.0–70.0%) (34). The role of FFM accretion may help interpret the correlations between weight gain and EI.

Specifically, we found that the magnitude of weight gain was correlated with MI, whereas FM was not significantly correlated with either SMR or weight gain. The implication is therefore that deposition of new fat-free tissue may be an additional stimulant to appetite, alongside baseline FFM and RMR. Consistent with that hypothesis, we found that mean parental height, a marker of the infant's “growth drive,” was associated with MI, EI, and weight gain, though not with FFM, after correcting for multiple comparisons. These associations suggest that the expression of the infant's growth drive is another physiological factor stimulating appetite. Our use of mean parental height as a marker of infant growth drive was based on previous studies showing similar associations of maternal and paternal height with infant weight gain (24, 25), and high heritability of postnatal weight gain (23). Since infant appetite has also been shown to be heritable (47) and since shared genetic effects may underpin associations of infant appetite and weight gain (48), future work could test whether shared genetic effects relate parental anthropometry to offspring appetite.

Collectively, these findings, along with the body of evidence on adults, suggest that this could be a fundamental process underlying appetite control in humans. From an evolutionary perspective, it makes sense that food (energy) intake is driven to satisfy active metabolic energy demands. This body of evidence provides a framework for thinking about human appetite control in which the behavior of EI is a response to EE, rather than a device for regulating adipose tissue stores.

Although there is concern that rapid infant weight gain may increase the risk of obesity at later ages (49–51), characterizing excess weight gain during infancy itself is difficult. Indeed, it might be assumed that excess weight gain is not possible during the period of exclusive breastfeeding due to maternal constraints on milk production; however, recent studies have found that such excess weight gain does occur very rarely (52). Interestingly, nutritional analyses showed that the primary difference between a group of exclusively breastfed infants gaining weight very rapidly versus those growing normally was not the energy content of the milk consumed, but rather lower leptin content in the breast milk (53). The low leptin level may encourage the infant to suckle for longer, and hence override the drivers of appetite that we have associated here with SMR and body composition. Other evidence shows that excess weight gain in early life primarily occurs after the onset of complementary feeding (54). Collectively, this supports the hypothesis that the primary cause of excess weight gain lies in the composition of food, or the way in which it is offered to the infant, rather than an internal failure of appetite control.

While our findings complement those in adults, the same hypotheses should also be tested in other age groups. Particularly around the time of pubertal growth spurts, both sexes demonstrate prolonged positive energy balance, and fat accretion may occur in the absence of excess weight gain. Previous studies have linked energy intake with skeletal muscle mass in overweight adolescents (8) but with both FFM and FM in children (55). Conversely, experimentally imposed increases in physical activity levels did not increase energy intakes in younger children (56, 57). Thus, this conceptual framework may benefit from further investigation in older infants, children, and adolescents.

A strength of the study is that the data were collected before the UK obesity epidemic, in the early 1990s. The mean maternal BMI in our study sample was 23.2 kg/m^2^, and only 1 mother had a BMI >30 kg/m^2^. This suggests that there was little potential for maternal metabolic perturbations to impact infant appetites among those breastfeeding. Another strength is that, unlike food intakes in older age groups, the measurement of infant milk intake has relatively low levels of measurement error. We sampled milk intake on 2 days, and also validated our test-weighing results against isotopic measurements in the formula-fed infants (58). Moreover, except for body composition and TEE, all of the associations tested involved traits measured using independent techniques, such that there is no contribution of correlated error. Finally, our sample comprised both breastfed and formula-fed infants; hence, our results may generalize across infant feeding modes.

However, a limitation of our estimations of EI is that breast milk energy content can still vary between individual mothers, which we did not address. Another limitation comprises the small sample size, though we still detected several significant correlations that closely replicated previous findings in adults (7). Similarly, we studied only families of white European ethnicity. Our parental heights were obtained by self-report rather than direct measurement, but this may make our finding of a correlation of average parental height with infant weight gain conservative, as any recall error would likely reduce the magnitude of the correlation. Finally, as highlighted by Lam and Ravussin (42), it is still not clear what signals are emitted by metabolically active tissues or basal metabolic processes that regulate appetite and feeding behavior, and studies such as this cannot attribute causation.

In conclusion, our study replicates recent findings in adults by linking EI in 3-month-old infants strongly with FFM and SMR, but not with adiposity. What our study adds beyond previous work is that these relationships appear to persist at an age when positive energy balance and fat accretion are the norm. We suggest that this may be partly explained by the fact that even at this age the majority of weight gain comprises FFM accretion, which may further stimulate appetite to support such fat-free tissue growth. Together with similar work in adults, our findings indicate the operation of a fundamental physiological process of appetite regulation that is quite independent of environmental influences.

## Data Availability

Data described in the manuscript, code book, and analytic code will be made available upon request pending application.

## Notes

Data collection was supported by the UK Medical Research Council. This study benefitted from funding from the Medical Research Council to the Dunn Nutrition Unit.

Abbreviations used: BMR, basal metabolic rate; EE, energy expenditure; EI, energy intake; FFM, fat-free mass; FM, fat mass; MI, milk intake; RMR, resting metabolic rate; SMR, sleeping metabolic rate; TEE, total energy expenditure
